# Profile of mood states and stress-related biochemical indices in long-term yoga practitioners

**DOI:** 10.1186/1751-0759-5-6

**Published:** 2011-06-03

**Authors:** Kazufumi Yoshihara, Tetsuya Hiramoto, Nobuyuki Sudo, Chiharu Kubo

**Affiliations:** 1 Department of Psychosomatic Medicine, Graduate School of Medical Sciences, Kyushu University, 3-1-1 Maidashi, Higashiku, Fukuoka, Japan; 2 Division of Cerebral Integration, Department of Cerebral Research, National Institute for Physiological Sciences, 38 Myodaiji-Nishigonaka, Okazaki, Aichi, Japan

## Abstract

**Background:**

Previous studies have shown the short-term or intermediate-term practice of yoga to be useful for ameliorating several mental disorders and psychosomatic disorders. However, little is known about the long-term influences of yoga on the mental state or stress-related biochemical indices. If yoga training has a stress-reduction effect and also improves an individual's mental states for a long time, long-term yoga practitioners may have a better mental state and lower stress-related biochemical indices in comparison to non-experienced participants. This study simultaneously examined the differences in mental states and urinary stress-related biochemical indices between long-term yoga practitioners and non-experienced participants.

**Methods:**

The participants were 38 healthy females with more than 2 years of experience with yoga (long-term yoga group) and 37 age-matched healthy females who had not participated in yoga (control group). Their mental states were assessed using the Profile of Mood States (POMS) questionnaire. The level of cortisol, 8-hydroxydeoxyguanosine (8-OHdG) and biopyrrin in urine were used as stress-related biochemical indices.

**Results:**

The average self-rated mental disturbance, tension-anxiety, anger-hostility, and fatigue scores of the long-term yoga group were lower than those of the control group. There was a trend toward a higher vigor score in the long-term yoga group than that in the control group. There were no significant differences in the scores for depression and confusion in the POMS between the two groups. The urine 8-OHdG concentration showed a trend toward to being lower in the long-term yoga group in comparison to the control group. There were no significant differences in the levels of urine biopyrrin or cortisol.

**Conclusions:**

The present findings suggest that long-term yoga training can reduce the scores related to mental health indicators such as self-rated anxiety, anger, and fatigue.

## Background

Yoga is an ancient technique of promoting health through exercises, regulation of breathing, and meditation. The practice of yoga has increased in many countries, such as the United States (US), the UK, and Japan. The popularity of yoga in US adults is evident by the estimated increase in participation from 3.7% in 1997, 5.1% in 2002, to 6.1% in 2007 [1,2]. Yoga has been reported to have various therapeutic effects. Studies have shown that the practice of yoga reduces perceived stress and negative feelings and improves mental and physical symptoms [3-6]. Yoga has therapeutic benefits not only for various mental disorders [7-10] but also for some physical diseases, such as asthma [11], hypertension [12], rheumatoid arthritis [13], migraines [14], musculoskeletal disorders [15], cancer-related symptoms [16] and other disorders [17], most of which are related to mental factors or mental states, or are aggravated by stress.

It has been demonstrated that the practice of yoga improves the mental state by lowering the levels of anxiety, depression and anger [18-21]. Lavey et al. indicated that tension-anxiety, depression, anger-hostility, fatigue, and confusion in psychiatric inpatients were improved following at least one yoga session [19]. Manocha et al. indicated that a 4 month intervention with yoga reduced anger-hostility and fatigue scores in asthma patients [20]. Sareen et al. demonstrated that 12 weeks' intervention with yoga reduced tension-anxiety, depression, anger-hostility, fatigue, and confusion scores in chronic pancreatitis patients [21]. While these limited studies have revealed that yoga can improve the self-rated tension-anxiety, depression, anger-hostility, or fatigue scores, this improvement has only been shown after short-term or intermediate-term practice of yoga. The potential effects of long-term yoga practice on a person's mental state have not been investigated.

It is known that the mental state can influence the hypothalamic-pituitary-adrenal (HPA) axis and alter the cortisol levels. Cortisol, secreted from the adrenal cortex, is a biochemical index of HPA axis activation, and is an important marker related to psychological stress. Some studies have demonstrated that the long-term practice of meditation, which is one of the components of yoga, does not to lead to any significant difference in the baseline cortisol level between practitioners and control participants [22-24]. With regard to yoga, however, Vera et al. showed that there was a significantly higher level of baseline cortisol in long-term yoga practitioners than in the control participants [25].

Besides cortisol, there have been several studies comparing other biochemical indices of experienced meditation practitioners with those of less experienced practitioners, or non-experienced participants [24,26-29]. For example, catecholamine levels, such as norepinephrine, epinephrine and vanillylmandelic acid (VMA), are representative examples of stress-related biochemical indices. Lang et al. reported the daily urinary excretion of norepinephrine, epinephrine, and VMA to all be higher in qualified meditators compared with less experienced practitioners [26]. On the other hand, Infante et al. reported that the urinary levels of norepinephrine and epinephrine in the meditation group were lower than those in the control group [24,27], and Walton et al. found low concentrations of urinary VMA in meditation practitioners in comparison with nonpracticing participants [28]. Therefore, the impact of long-term medication on the catecholamine levels appears to be controversial, and further studies are needed to conclusively determine the impact of long-term meditation on stress levels.

Recently, urinary stress-related biochemical indices, such as 8-hydroxydeoxyguanosine (8-OHdG) and biopyrrin, have been used to assess the psychological stress because the blood sampling itself is invasive and can be associated with psychological stress [30-33]. The urinary 8-OHdG level is a putative biomarker of total systemic oxidative stress [34] and psychological distresses are associated with oxidative damage [30]. Biopyrrin, which is an oxidative metabolite of bilirubin, is also one of the urinary stress-related biochemical indices [31-33]. However, there have been no previous studies comparing the 8-OHdG or biopyrrin levels of experienced yoga practitioners with those of less experienced practitioners, or non-experienced participants.

If yoga training has a stress-reduction effect and also improves an individual's mental states for a long time, long-term yoga practitioners may have a better mental state, which would be indicated by lower scores for mental disturbance, anxiety, anger, depression, and fatigue, and lower stress-related biochemical indices in comparison to non-experienced participants. Therefore, the current study simultaneously examined the differences in mental states and urinary stress-related biochemical indices between long-term yoga practitioners and non-experienced participants. The purpose of this study was to examine the beneficial effects of long-term yoga on indicators of psychological distress, such as anxiety, anger, depression, and fatigue levels in the profile of mood states questionnaire (POMS), and on the stress-related biochemical indices, such as biopyrrin and 8-OHdG levels, in healthy individuals.

## Methods

### Participants and data collection

This study evaluated thirty-eight healthy, adult females with more than 2 years of experience with yoga (long-term yoga group: we defined more than 2 years of experience as being long-term in this study) and 37 age-matched healthy, adult females who had no experience with yoga (control group). The participants in the long-term yoga group were recruited from qualified yoga instructors and their students who had more than 2 years of yoga experience from ten yoga training centers in Fukuoka, Kumamoto, and Kagoshima prefectures of Japan. The control group participants were recruited from among the acquaintances of the qualified yoga instructors in the same prefectures. The following exclusion criteria were applied to participants: (i) age < 20 years old or > 60 years old; (ii) taking medication including supplements in the last 1 month prior to the experiment; (iii) having an illness; or (iv) having a past history of a physical or mental illness. All participants received 500 yen (about $5.50) for participating in this study. All participants received detailed information on the purpose about the study and provided written informed consent. The participants who agreed were then handed two questionnaires, a paper cup, a tube with a screw cap, and a self-addressed parcel. Each participant collected her urine at home and she answered the questionnaires at the same time. The urine and the questionnaires was sent via a parcel delivery service, which used a freezer van to keep the samples frozen at -18°C (-0.4°F), as soon as possible. This study was approved by the Institutional Review Board at Kyushu University.

### Yoga in the long-term yoga group

The long-term yoga group participants had practiced yoga in sessions of an average of 2.94 ± 2.04 days a week (Range: 1-7), with a duration of 1.37 ± 0.49 hours each day (Range: 0.5-3.0), for 3.70 ± 2.27 years (Range: 2-14) on average. The calculated mean total hours of yoga exercise using these values was 1174 ± 2243 (Range: 104-13104). They practiced the structured classic postures (Shavasana, Tadasana, Ardha Kati Chakrasana, Pada Hastasana, Ardha Chakrasana, Shashankasana, Ardha Ushtrasana, etc.), breathing exercises (Kapalabhati Pranayama, Anuloma Pranayama, Bhramari Pranayama, etc.), and meditation (Om meditation etc.). In addition, most of them mainly practiced the structured classic postures.

### Urine sampling

Urine samples were taken from all participants for the quantification of biopyrrin, 8-OHdG, cortisol and creatinine. The participants were asked to avoid vigorous exercise and heavy psychological stress for 24 hours prior to urine collection. Urine was collected at 6:00-9:00 am, and 2 ml of urine from each participant was stored at -80°C until it was analyzed. The urinary biopyrrin, cortisol, and 8-OHdG concentrations were measured by enzyme-linked immunosorbent assay kits (Shino-Test, Tokyo, Japan, Oxford Biomedical Research, Inc. MI, and Nikken Seil Co., Ltd, Shizuoka, Japan, respectively). The urine creatinine concentration was analyzed using the Accuras Auto-Cre diagnosis kit (Shino-Test, Tokyo, Japan) and biopyrrin, 8-OHdG and cortisol concentrations were corrected based on the urine creatinine concentration.

### Questionnaire

A self-completed questionnaire about demographic and yoga-related characteristics, and the POMS (Educational and Industrial Testing Service, San Diego, CA) questionnaire were given to all of the participants. The self-completed questionnaire included questions about age, race, education, mean number of hours per day and mean number of days per week, spent practicing yoga at home or in a yoga center, and years of yoga experience. The POMS questionnaire assesses six mood subscales: tension-anxiety, depression, anger-hostility, vigor, fatigue, and confusion. High vigor scores reflect a good mood or emotion, and low scores in the other subscales reflect a good mood or emotion. The total mood disturbance score was computed by adding the five negative subscale scores (tension-anxiety, depression, anger-hostility, vigor, fatigue, and confusion) and subtracting the vigor score. Higher scores for the total mood disturbance score indicate a greater degree of mood disturbance. These scores have been widely used in the assessment of mood states. Yokoyama et al. previously translated the 65-item scale in the POMS into Japanese, and demonstrated the reliability and validity of the Japanese version of the POMS in Japanese participants [35]. The Japanese version of the POMS (Kaneko Shobo Co., Tokyo, Japan) was used for the present study.

### Statistical analysis and sample size

Statistical analyses were performed by using a statistical software package (PASW Statistics 18, version 18.0.0 for Windows; SPSS Inc., Chicago, IL, USA). The unpaired *t*-test and the Pearson's chi-square test were used to compare the age and education between the long-term yoga group and the control group, respectively. The Mann-Whitney U-test was used to compare the biopyrrin, 8-OHdG and cortisol concentrations, which were corrected based on the urine creatinine concentration, and the scores of questionnaires between the long-term yoga group and the control group. The Spearman rank correlation was used to test the relationship between the total hours of yoga exercise and the POMS scores. Differences of *p *< 0.05 were considered to be statistically significant.

We estimated that a sample size of 35 in each group would allow us to detect any difference between the groups in some POMS scores with 80% power (α = 0.05) based on the mean and standard deviation of each POMS score reported in a previous study [21]. This sample size would also be sufficient to detect a meaningful difference in cortisol between the groups with reference to the sample size of the previous study [25].

## Results

### Participant demographics

The demographic data of the participants including their age, race, and education are shown in Table 1. None of these variables were significantly different between the control and the long-term yoga groups.

**Table 1:** Demographic of the control and long-term yoga groups

Characteristics	Control group (n = 37)	Long-term yoga group (n = 38)	Statistic	*p*-value
Age [Mean (standard deviation)]	34.43 (8.16)	33.84 (7.33)	t = 0.330	0.743
Range	(22-49)	(22-49)		
Race (%)				
Asian	100 (n = 37)	100 (n = 38)		
Education (%)			χ² = 0.958	0.812
Junior H.S. graduate	0.0 (n = 0)	2.6 (n = 1)		
H.S. graduate	24.3 (n = 9)	23.7 (n = 9)		
Junior college graduate	37.8 (n = 14)	42.1 (n = 16)		
College graduate	29.7 (n = 11)	31.6 (n = 12)		
Blank	8.1 (n = 3)	0.0 (n = 0)		

The unpaired *t*-test and Pearson's chi-square test were used to compare the age and education levels between the long-term yoga group and control group, respectively.

### Profile of mood states

There were some significant differences between the control and the long-term yoga group in the POMS scores (Table 2). The long-term yoga group showed a lower total mood disturbance (*p *= 0.015), tension-anxiety (*p *= 0.017), anger-hostility (*p *= 0.007), and fatigue score (*p *= 0.008) in the POMS. There was a trend toward a higher vigor score in the long-term yoga group than in the control group (*p *= 0.082). There were no significant differences in the scores for depression and confusion in the POMS.

**Table 2:** Scores of Profile of Mood States (POMS) in the control and long-term yoga groups

Variable	Control group	Long-term yoga group
Profile of Mood States (POMS)		
Total mood disturbance score	34.11 ± 31.46	18.03 ± 31.54 *
Tension-Anxiety	9.86 ± 6.01	6.95 ± 5.84 *
Depression	9.81 ± 8.52	7.89 ± 9.46
Anger-Hostility	10.08 ± 7.40	6.29 ± 6.97 **
Vigor	12.16 ± 5.59	15.00 ± 6.57
Fatigue	8.65 ± 5.94	5.47 ± 5.31 **
Confusion	7.86 ± 4.73	6.42 ± 4.68

Results are presented as the means ± standard deviation (SD). *: *p *< 0.05; **: *p *< 0.01

### Stress-related biochemical indices

The urine 8-OHdG concentration showed a trend toward being lower in the long-term yoga group in comparison to the control group (*p *= 0.067, Table 3). There were no significant differences in the levels of urine biopyrrin or cortisol (Table 3).

**Table 3:** Urine concentrations of cortisol, 8-hydroxydeoxyguanosine (8-OHdG), and biopyrrin in the control and long-term yoga groups

Variable	Control group	Long-term yoga group
Urine concentration		
Cortisol (μg/gCre.)	0.951 ± 0.457	0.803 ± 0.358
8-OHdG (μg/gCre.)	0.125 ± 0.061	0.109 ± 0.080
Biopyrrin (u/gCre.)	1.216 ± 0.410	1.266 ± 0.736

Results are presented as the means ± standard deviation (SD).

### Correlation between the total hours of yoga exercise and the POMS scores in the long-term yoga group

To investigate the "dose" effect on the POMS scores by the total hours spent in yoga exercise, we calculated the correlation between the total hours of yoga exercise and the mental disturbance, tension-anxiety, anger-hostility, and fatigue scores on the POMS in the long-term yoga group. However, no correlations were identified between them (data not shown).

## Discussion

The present study demonstrated that long-term yoga practitioners have lower mental disturbance, tension-anxiety, anger-hostility, and fatigue scores in the POMS test in comparison to non-experienced participants, although there were no significant differences in the levels of urinary stress-related markers. This is the first study to demonstrate that long-term yoga practitioners have a better mental state than healthy participants who do not engage in yoga. These findings suggest that ongoing yoga training reduces the level of mental disturbance, anxiety, anger, and fatigue not only over the short- or intermediate-term, but also over a long term.

Yoga was found to be as effective as relaxation in reducing anxiety [5]. A systematic review of the use of yoga to treat anxiety suggested that yoga may be beneficial for different anxiety disorders [4]. Waelde et al. indicated that an intervention consisting of a six-session yoga program reduced the anxiety of dementia caregivers who did not have an anxiety disorder [36]. In addition, a week of yoga practice prevented an increase in anxiety among flood survivors [37]. Moreover, significant reductions were shown for anger and anxiety symptoms in participants who had been diagnosed with unipolar major depression in partial remission and who completed 20 yoga sessions [9]. These reports and the present results suggest that the anxiolytic and relaxational effects of yoga may continue for an extended period.

Regarding the level of depression in the current study, there was no significant difference between the depression score in the long-term yoga group and that in the control group, which was unexpected. Previous studies of yoga for depression suggest that yoga may have beneficial effects not only among depressive patients [9], but also non-patients with elevated symptoms of depression [38,39]. However, the current results indicated that the mean depressive scores of both the long-term yoga group and the control group were lower than 11, which is equivalent to 50 based on T-score conversion (the T-score table is attached to the Japanese version of the POMS). Therefore, it may be difficult for non-depressive healthy participants to reduce their depressive score in the POMS. Like the mean depressive scores, the mean confusion scores in both groups were lower than 8.5, which is equivalent to 50 based on the T-score conversion. Therefore, there was limited potential for there to be any change in the confusion score in the POMS.

On the other hand, we could not find a "dose" effect for yoga with regard to the mental disturbance, tension-anxiety, anger-hostility, and fatigue scores on the POMS in the long-term yoga group. The mean scores in the long-term yoga group were lower than those in the control group, but the scores in the control group were lower than the equivalent values of 50 based on the T-score conversion, suggesting the presence of a floor effect. In other words, it is speculated that the scores of mental disturbance, tension-anxiety, anger-hostility, and fatigue on the POMS may not decrease after a certain point within 2 years, even if practitioners continue to exercise yoga. A longitudinal study is needed to verify this speculation.

As for urinary 8-OHdG, previous studies have shown that a depressive state affects female 8-OHdG levels in leukocytes [40,41]. Forlenza and Miller also reported that severely depressed individuals had increased serum levels of 8-OHdG in comparison to healthy individuals [42]. In the present study, the urinary 8-OHdG level in the long-term yoga group tended to be lower than that in the control group, although there was no significant difference between the depression score of the POMS in the long-term yoga group and that in the control group. Psychological distress, not depression, may be important to increasing the concentration of urinary 8-OHdG. Further studies are needed to clarify the relationship between 8-OHdG and psychological distress.

Concerning urinary biopyrrin, several studies have indicated that the urinary excretion of biopyrrins is higher in patients with psychiatric disorders, such as schizophrenia [31,32] and it increases due to psychological stress [33]. However, the current results showed no significant difference between the urinary biopyrrin levels of the long-term yoga group and that of the control group. This may be because it is difficult for healthy people to reduce their concentration of urinary biopyrrin by yoga training because it would appear that their urinary excretion of biopyrrins, in healthy people, is already stable at a lower concentration.

Cortisol is an accepted objective stress-related biological marker, because dysregulation of the level of cortisol is related to pathologies associated with stress-related symptoms, such as anxiety, depression, and negative affect [43-45]. The decrease in cortisol soon after yoga practice shown by West et al. [46] was similar to that seen in many types of interventions to reduce stress, such as meditation [22,47,48] and progressive muscle relaxation [49]. On the other hand, Vera et al. showed that a long-term yoga group displayed higher baseline cortisol levels than control participants [25]. This result was inconsistent with the present result which showed that the baseline cortisol level of the long-term yoga group was not significantly different from that of the control group. As both Vera et al.'s study and our study are cross-sectional studies, further research is needed to conclusively determine how baseline cortisol levels change over time in long-term yoga practitioners.

There are several limitations in this study. First, a relatively small number of participants were examined in this study. However, we were still able to demonstrate significant differences in some POMS scores because we properly estimated the sample size. Second, the POMS scores, some of which seemed to be influenced by the menstrual cycle, and stress-related biochemical indices were assessed only once throughout the study. Third, it was impossible to discriminate whether the practice of yoga improved the participant's mental state, or whether practitioners who had a good mental state continued to practice yoga, because this study was a cross-sectional study. Although the results must be interpreted with caution, it is possible to conclude that the improvement of mental health with yoga may continue for an extended period, because there is ample evidence that short-term or intermediate-term practice of yoga improves the participants' mental status. The fourth limitation is that the participants were only females. The long-term practice of yoga may have little effect on males. However, mood improvements by yoga are probably not related to gender in healthy participants, because Lavey et al. demonstrated that mood improvement with yoga practice was not related to gender in psychiatric inpatients [19]. Fifth, the comparison of baseline demographics is limited. We did not measure the current psychological stress levels except by using the POMS, or current exposure to situations likely to cause increased or decreased stress, such as lifestyle, work situation, any other forms of physical exercise or relaxation techniques. Also, we did not confirm whether the total hours of yoga exercise in the long-term yoga group were true, because this information was self-reported, and whether participants had actually avoided vigorous exercise and heavy psychological stress for 24 hours prior to testing. The sixth limitation is that we defined more than 2 years of experience as long-term in this study. Further research is needed to elucidate the far longer-term effects of yoga practice. Finally, the current study measured urinary biopyrrin and baseline cortisol concentrations as stress-related biochemical indices, which are unsuitable or insufficient to assess the mental state of healthy individuals. A multifaceted approach combining the assessment of more suitable stress-related biological markers such as the immune, endocrine, or autonomic nervous systems, and brain function, are important to more accurately assess the mental state of healthy individuals.

Despite these limitations, the present findings suggest that long-term yoga training can reduce the score related to mental health indicators such as self-rated anxiety, anger, and fatigue. Long-term yoga training may affect mental well-being and be useful to prevent mental disorders over an extended period. Further studies that measure self-rated mental states, stress-related biomarkers of the immune, endocrine, autonomic nervous systems, and brain function simultaneously are needed to verify the beneficial effects of yoga and to elucidate the mechanism(s) responsible for the benefits that are associated with yoga.

## Conclusion

The present findings suggest that long-term yoga training can reduce the scores related to mental health indicators such as self-rated anxiety, anger, and fatigue.

## Abbreviations

POMS: Profile of Mood States; 8-OHdG: 8-hydroxydeoxyguanosine; HPA: hypothalamic-pituitary-adrenal; VMA: vanillylmandelic acid; SD: standard deviation.

## Competing interests

The authors declare that they have no competing interests.

## Authors' contributions

KY conceived the study, participated in the design of the study, carried out data collection, performed the statistical analysis and drafted the manuscript. TH participated in the design of the study and carried out data collection. NS evaluated the results of the study and reviewed the manuscript. CK participated in the design and coordination of the study and reviewed the manuscript. All authors read and approved the final manuscript.
